# Differential Impact of IL-10 Expression on Survival and Relapse between HPV16-Positive and -Negative Oral Squamous Cell Carcinomas

**DOI:** 10.1371/journal.pone.0047541

**Published:** 2012-10-31

**Authors:** Chun-Yi Chuang, Wen-Wei Sung, Lee Wang, Wea-Long Lin, Kun-Tu Yeh, Mao-Chang Su, Chun-Han Hsin, Shiann-Yann Lee, Buor-Chang Wu, Ya-Wen Cheng, Huei Lee

**Affiliations:** 1 Institute of Medicine, Chung Shan Medical University, Taichung, Taiwan, Republic of China; 2 Institute of Medical and Molecular Toxicology, Chung Shan Medical University, Taichung, Taiwan, Republic of China; 3 School of Medicine, Chung Shan Medical University, Taichung, Taiwan, Republic of China; 4 Department of Pathology, Chung Shan Medical University, Taichung, Taiwan, Republic of China; 5 Department of Otolaryngology, Chung Shan Medical University Hospital, Taichung, Taiwan, Republic of China; 6 Department of Oral and Maxillofacial Surgery, Chung Shan Medical University Hospital, Taichung, Taiwan, Republic of China; 7 Department of Pathology, Changhua Christian Hospital, Changhua, Taiwan, Republic of China; 8 Graduate Institute of Cancer Biology and Drug Discovery, Taipei Medical University, Taipei, Taiwan, Republic of China; 9 School of Public Health, Chung Shan Medical University, Taichung, Taiwan, Republic of China; National Taiwan University Hospital, Taiwan

## Abstract

Human papillomavirus (HPV) is a risk factor in a subset of oropharyngeal cancer; however, the contribution of HPV in the malignancy of oral squamous cell carcinomas (OSCC) is not fully understood in Taiwanese. Herein, 61 patients with no risk factors and 117 patients with one or more risk factors were enrolled in this study. HPV16/18 infection rate in non-smokers, non-drinkers and non-betel quid chewers was higher than their counterparts. The development of HPV-infected cancer has been shown to be associated with interleukin-10 (IL-10) expression. To this end, IL-10 mRNA expression in OSCC tumors was evaluated by real-time RT-PCR. Data showed that HPV-positive patients had higher IL-10 mRNA levels than in HPV-negative patients. Kaplan-Meier and Cox-regression analysis indicated that the prognostic significance of IL-10 mRNA on overall survival and relapse free survival was only observed in HPV-positive OSCC, but not in HPV-negative OSCC. Mechanistically, the elevation of IL-10 by E6 was responsible for increased colony formation and migration capability in OSCC cells. Therefore, we suggest that IL-10 induced by E6 promotes cell growth and migration capability and consequent poor survival and relapse in HPV-positive OSCC.

## Introduction

Oral squamous cell carcinomas (OSCC) arise from the mucosa of the oral cavity including border of tongue, buccal cavity, palate, gingival and lip. This disease is the tenth most common cancer in the world and is the fourth most common cancer among men in Taiwan [1]. However, the disease is rare among women in Taiwan, possibly because of etiological factors such as cigarette smoking, alcohol drinking, and betel quid chewing habits that are predominately observed in men and rarely in women [2]–[4]. A subset of oropharyngeal cancer including pharynx, tonsil, and base of tongue of non-smokers and non-drinkers in the Caucasian and Chinese population has been shown to be linked to human papillomavirus (HPV) infection, but the contribution of HPV in the malignancy of oral squamous cell carcinomas (OSCC) is not fully identified in Taiwanese [5]–[15]. Therefore, we hypothesized that HPV infections might play a role in the development of OSCC in members of the Taiwanese population who do not have the habits of alcohol drinking, betel quid chewing, or cigarette smoking [16]. In addition, HPV16-positive OSCC has been shown to have a more favorable outcome than HPV16-negative OSCC [7], [17]–[23]. However, HPV16 infection in advanced oral cavity cancer patients is related to an increased risk of distant metastases and poor survival [24].The reason remains unclear, but may involve interactions of cytokines such as interleukin-10 (IL-10) which have been shown to associate with viral-infected cancer development [25]–[28].

IL-10 is an important immunoinhibitory cytokine that is part of a balanced cytokine network [29], [30]. It is produced by several cells including normal and neoplastic B cells, stimulated monocytes/macrophages, a subset of T cells, and some cancer cells [30]–[32]. Brook et al. showed that persistent viral infection in mice results in a significant upregulation of IL-10 by antigen-presenting cells, and leads to impaired T-cell responses [28]. Genetic removal of IL-10 resulted in the rapid elimination of the virus and the development of antiviral memory T-cell responses [28]. Therefore, IL-10 has been documented to determine *in vivo* virus clearance or persistent infection. Previously, IL-10 expression levels were reported as significantly higher in an HPV16-infected high grade of cervical intraepithelial neoplasia (CIN) II and III when compared to normal cervical epithelium and CIN I [33]–[35]. This suggests that IL-10 production may play a role in the progression of HPV-associated cervical precancer [34].

In the present paper, 61 patients with no risk factors and 117 patients with one or more risk factors (smoking, drinking, and betel quid chewing) were enrolled to explore: (I) whether HPV16/18 infection may be a predominant etiological factor for OSCC of non-smokers, non-drinkers, and non-betel quid chewers when compared with smokers, drinkers, and betel quid chewers; and (II) whether IL-10 expression might play a more important role in the progression of HPV16/18-positive OSCC than in HPV16/18-negative OSCC and consequently would result in poor overall survival (OS) and relapse free survival (RFS) in these patients.

## Materials and Methods

### Study subjects

OSCC tumor specimens were collected between 2001 and 2010 from 178 patients with primary oral cancers in the Department of Otolaryngology, Chung-Shan Medical University Hospital (Taichung, Taiwan) and the Department of Surgical Pathology, Changhua Christian Hospital (Changhua, Taiwan). This study is approved by the Institutional Review Board, Chung Shan Medical University Hospital (CSMUH No: CS11178; informed consent form: waiver). The tumor type and stage of each collected specimen were histologically determined according to the WHO classification system. Cancer relapse data were obtained by chart review and confirmed.

### Cell lines

The SiHa (cervical cancer) [36], SAS, TW2.6, HSC3, GNM, SCC4, and SCC25 oral cancer cell lines were maintained in DMEM. Cell lines were kindly provided by Dr. T. C. Lee, Institute of Biomedical Sciences, Academia Sinica, Taipei, Taiwan [37]. The GNM cell line was kindly provided by Dr. M. Y. Chou, Department of Dentistry, Chung Shan Medical University, Taichung, Taiwan [38]. The medium contained 10% fetal bovine serum supplemented with penicillin (100 U/ml) and streptomycin (100 mg/ml). Cells were grown at 37°C in a humidified incubator at 5% CO_2_.

### Nested polymerase chain reaction (Nested-PCR)

Tumor genomic DNA was extracted from the tumor portion of whole-mount paraffin sections of OSCC specimens. SiHa and HeLa cervical cancer cells were used as positive controls for the detection of HPV16 and HPV18 DNA, and PBS was used as a negative control. HPV viral DNA was first amplified with type consensus primers MY09 and MY11 followed by a second round of amplification with type-specific primers flanking the L1 region to identify the subtype. The detailed procedures were described previously [39].

### Plasmid construction and transfection reaction

Different concentrations of HPV16 E6 plasmids were transiently transfected into oral cancer cells (1×10^6^) using the TurboFect reagent (Fermentas) as described previously [40].

### Silencing of HPV-18 E6 and IL-10 expression by RNA interference

The RNA interference target sequences for HPV18 E6 shRNA have been previously verified [41]. IL-10 shRNA was purchased from National RNAi Core Facility, Academia Sinica, Taiwan. Detailed procedures were described previously [40].

### RNA isolation and real-time RT-PCR

Total RNA was extracted from the tumor portions of whole-mount paraffin sections of OSCC specimens, by TRIzol reagent chloroform extraction and isopropanol precipitation. Total RNA from tumor tissues was transcribed using a High Capacity cDNA Reverse Transcription Kit (Applied Biosystems, ABI) to obtain a cDNA library [40]. Real-time RT-PCR primers were as follows: for IL-10 transcripts, 5′-GGCGCTGTCATCGATTTCTT-3′ (forward) and 5′-TGGAGCTTATTAAAGGCAT -TCTTCAC-3′ (reverse); for 18S gene transcripts, 5′-TCGGAACTGAGGCC -ATGA-3′ (forward) and 5′-CCGGTCGGCATCGTTTA-3′ (reverse), and the reactions were performed as described previously [40], [41]. The PCR product of IL-10 was confirmed by direct sequencing.

### Protein extraction and Western blot

Anti-IL10 (E-10; Santa Cruz Biotechnology), anti-HPV16 E6 (N-17; Santa Cruz Biotechnology) and anti- HPV18 E6 (G-7; Santa Cruz Biotechnology) primary antibody were used in western blot. The procedures were described previously [41].

### Colony formation and migration assay

Transfected cells were seeded into 6-well plate (100 cell per well) and, then, fixed and stained with 10% Geimsa after a ten-day colony formation period. Cell migration ability was measured by Boyden chamber assay with an 8 µm pore polycarbonate membrane, according to the manufacturer's protocol. Transfected cells were fixed and stained with crystal violet after a 24-hour migration period.

### Immunohistochemical staining

Immunohistochemical staining to evaluate p16 expression in tumor tissue was performed on whole-mount paraffin sections of OSCC specimens. Anti-p16 (1/200) polyclonal primary antibody (Santa Cruz Biotechnology, USA) was used. An immunohistochemistry (IHC) detection kit for in vitro diagnostic use (Invitrogen, USA) was used according to the standard protocol. IHC staining scores were defined as previously described [40].

### Statistical analysis

The Student's t-test and the Chi-square test were applied for continuous or discrete data analysis, respectively. The associations between IL-10 expression and patient survival were estimated using the Kaplan-Meier method and assessed using the log-rank test. Potential confounders including gender, age and stage were adjusted by Cox regression models, with IL-10 expression fitted as indicator variables. Interaction was further assessed using the likelihood ratio test to calculate χ^2^ and p values. In the test for interaction, the Cox regression model with only main effects was compared to that with both main effect terms and interaction term. Interaction effect was defined as the difference of their deviance All statistical analyses were conducted using the SPSS statistical software program (version 13.0) (SPSS, Inc., Chicago, IL). All statistical testing was performed using two-sided tests and P values<0.05 were considered to be statistically significant.

## Results

### HPV 16/18 infections in Taiwanese OSCC patients are more common in females, non-smokers, non-drinkers, and non-betel quid chewers

We explored whether HPV16/18 infections in OSCC are more prevalent in non-smokers, non-drinkers, and non-betel quid chewers in Taiwan. A total of 178 OSCC patients were enrolled to determine the type of HPV16 and HPV18 DNA by nested PCR. As shown in Table 1, HPV16, HPV18, and combined HPV16/18 (HPV16/18) infection rates in females, non-smokers, non-drinkers, and non-betel quid chewers were higher than in males, smokers, drinkers, and betel quid chewers. HPV16 and HPV16/18 infections were more common in patients with OSCC of the tongue than in OSCC of the buccal or other areas (e.g., palate, gingiva, and lip). Surprisingly, a more than 70% HPV16/18 infection was found in females, non-smokers, and non-betel quid chewers. This finding suggests that HPV16/18 infections may play a crucial role in cancer development in this subset of OSCC patients.

**Table 1:** Relationships between HPV16, HPV18 and HPV16/18 infection and clinical parameters in OSCC patients.

Parameters	Case No.	HPV 16	HPV 18	HPV 16/18
Age				
<56	105	30 (28.6)	30 (28.6)	50 (47.6)
≥56	73	24 (32.9)	24 (32.9)	44 (60.3)
Gender				
Female	70	34 (48.6)*	31 (44.3)*	56 (80.0)*
Male	108	20 (18.5)	23 (21.3)	38 (35.2)
Smoking				
No	73	33 (45.2)*	30 (41.1)*	53 (72.6)*
Yes	105	21 (20.0)	24 (22.9)	41 (39.0)
Drinking				
No	99	35 (35.4)	39 (39.4)*	64 (64.6)*
Yes	79	19 (24.1)	15 (19.0)	30 (38.0)
Betel quid				
No	80	33 (41.3)*	36 (45.0)*	60 (75.0)*
Yes	98	21 (21.4)	18 (18.4)	34 (34.7)
Stage				
I+II	103	31 (30.1)	26 (25.2)	50 (48.5)
III+IV	75	23 (30.7)	28 (37.3)	44 (58.7)
T value				
T1+T2	135	40 (29.6)	40 (29.6)	69 (51.1)
T3+T4	43	14 (32.6)	14 (32.6)	25 (58.1)
N value				
N0	128	39 (30.5)	34 (26.6)	64 (50.0)
N1+N2	50	15 (30.0)	20 (40.0)	30 (60.0)
Tumor site				
Tongue	92	35 (38.0)*	35 (38.0)	60 (65.2)*
Buccal	68	17 (25.0)	15 (22.1)	28 (41.2)
Other^1^	18	2 (11.1)	4 (22.2)	6 (33.3)
IL10				
Low	89	18 (20.2)*	21 (23.6)#	34 (38.2)*
High	89	36 (40.4)	33 (37.1)	60 (67.4)

1 Other tumor sites includes plate (n = 1), gingival (n = 8), and lip (n = 9).

* p<0.05;

# p = 0.05.

P16 expression has been documented as a surrogate marker of HPV infection [5]. In the present study, 147 out of 178 OSCC paraffin sections were available for evaluation of p16 expression by immunohistochemical analysis. A prevalence of OSCC with p16-positive immunostaining was found and positive staining was significantly higher in HPV16, HPV18, and HPV16/18-positive tumors compared with their counterparts (Table S1). This result seems to confirm the detection of HPV16/18 DNA in OSCC by nested PCR in this study.

### Higher IL-10 mRNA expression levels are seen in HPV16, HPV18, and HPV16/18-positive OSCCs than in HPV-negative OSCCs

A link has been shown between IL-10 expression and HPV-infected CIN progression [35]. Therefore, we expected to find differential IL-10 mRNA expression levels between HPV-positive and -negative OSCC. The IL-10 mRNA expression levels in OSCC, evaluated by real-time RT-PCR, ranged from 0∼1402.2. A median value of 2.38 was used as a cut-off point to divide OSCC into low and high groups (low: 0.6±0.8, 0.0∼2.38; High: 237.0±413.5, 2.39∼1402.2). The IL-10 mRNA expression levels were not associated with any clinico-pathological parameters except for age: the prevalence of high IL-10 mRNA was greater in older patients (>56 years of age) than in younger patients (58.9% vs. 43.8%, P = 0.048; Table S2). Interestingly, the prevalence of high IL-10 mRNA was also greater in OSCC patients with HPV16, HPV18, and HPV16/18 infections than without HPV16, HPV18, and HPV16/18 infections. The odds ratios of HPV16-, HPV18-, and HPV16/18-positive OSCC were 2.679, 1.908, and 3.347, respectively, when compared with HPV16-, HPV18-, and HPV16/18-negative OSCC (Table 2). These results suggest that HPV16/18 infection could elevate IL-10 transcription in OSCC patients.

**Table 2:** Comparison of IL-10 mRNA expression levels between HPV-positive and HPV–negative OSCC patients.

		IL-10 mRNA			
HPV type	Case No.	Low (%)	High (%)	Odds Ratio (95% CI)	P value
HPV 16					
Negative	124	71 (57.3)	53 (42.7)	Referent	
Positive	54	18 (33.3)	36 (66.7)	2.679 (1.373–5.227)	0.003
HPV 18					
Negative	124	68 (54.8)	56 (45.2)	Referent	
Positive	54	21 (38.9)	33 (61.1)	1.908 (0.995–3.661)	0.050
HPV 16/18					
Negative	84	55 (65.5)	29 (34.5)	Referent	
Positive	94	34 (36.2)	60 (63.8)	3.347 (1.808–6.196)	<0.001

Fourteen of 178 patients had both HPV16 and 18 infections.

IL-10 low: 0.6±0.8 (0.0–2.38); IL-10 high: 237.0±413.5 (2.39–1402.2).

### IL-10 mRNA expression predicts OS and RFS in HPV-positive OSCC, but not in HPV-negative OSCC

**Figure 1:**
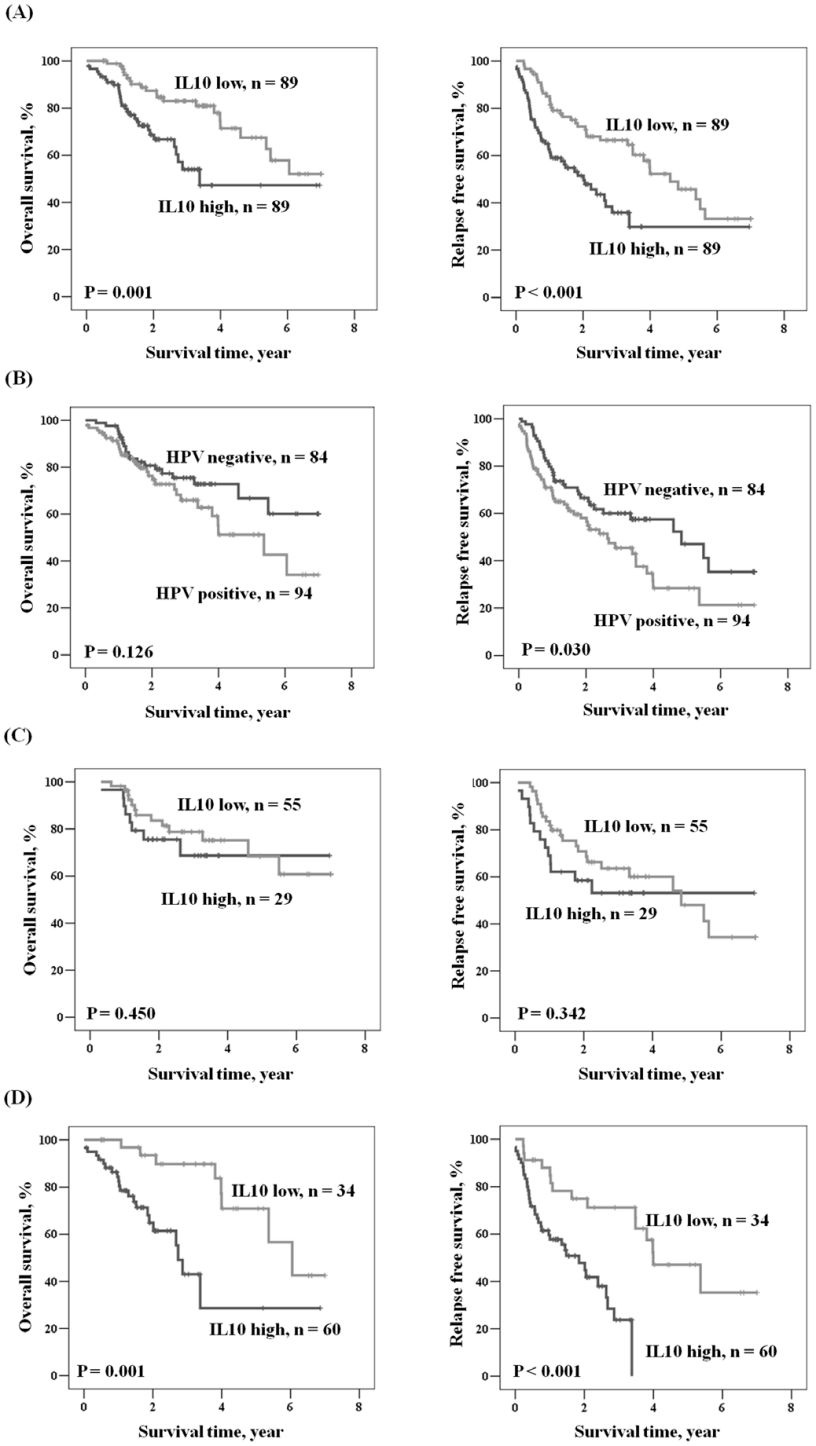
Kaplan-Meier analysis was used to assess the influence of IL-10 on OS and RFS. (A) in all study population, (B) in patients with HPV 16/18 infection compared to those without HPV 16/18 infection, (C) in patients with HPV 16/18 non-infection, and (D) in patients with HPV16/18 infection.

IL-10 has been shown to promote tumor progression via suppressed tumor immune surveillance [25], [32]. We therefore expected that IL-10 might be associated with the clinical outcome in HPV-positive OSCC. Kaplan-Meier analysis showed that patients with high IL-10 mRNA had shorter OS and RFS periods than did those with low IL-10 mRNA (P = 0.001 for OS; P<0.001 for RFS; Fig. 1A). Patients with HPV-positive tumors had shorter RFS when compared with those with HPV-negative tumors (P = 0.030), but a prognostic significance of HPV was not observed for OS (P = 0.126; Fig. 1B). More interestingly, the prognostic significance of IL-10 mRNA for OS and RFS was only shown in HPV-positive OSCC patients (Fig. 1D; P = 0.001 for OS; P<0.001 for RFS), but not in HPV-negative OSCC patients (Fig. 1C). Multivariate logistic regression analysis further indicated that patients with high IL-10 mRNA had hazard ratios (HR) of 2.438 and 2.098 for OS and RFS, respectively, when compared with patients with low IL-10 mRNA (95% CI, 1.345–4.419, P = 0.003 for OS; 95% CI, 1.329–3.311, P = 0.001 for RFS; Table 3). The five-year survival rate in patients with high IL-10 mRNA was lower than for patients with low IL-10 mRNA (47.2% vs. 67.4% for OS; 29.9% vs. 45.7% for RFS). Among HPV-positive OSCC patients, those with high IL-10 mRNA had HR of 4.810 and 3.807 for OS and RFS, respectively, when compared with patients with low IL-10 mRNA (95% CI, 1.946–11.892 for OS; 95% CI, 1.771–8.184 for RFS; Table 3). The five-year survival rate was significantly lower in patients with high IL-10 mRNA than with low IL-10 mRNA (28.7% vs. 70.8% for OS, P = 0.001; 0.0% vs. 47.0% for RFS, P = 0.001). As expected, we did not observe any prognostic significance of IL-10 mRNA in the entire study population or in HPV-negative patients (Table 3).

**Table 3:** Multivariate analysis of the influence of HPV 16/18 expression and IL-10 mRNA expression on overall survival (OS) and relapse free survival (RFS) in oral cancer patients.

Parameter	Case No.	OS				RFS			
		5-year survival (%)	HR	95% CI	P	5-year survival (%)	HR	95%CI	P
**IL-10**									
Low	89	67.4	1.000	Referent		45.7	1.000	Referent	
High	89	47.2	2.438	1.345–4.419	0.003	29.9	2.098	1.329–3.311	0.001
**HPV**									
Negative	84	72.6	1.000	Referent		49.2	1.000	Referent	
Positive	94	46.3	1.765	0.790–3.945	0.166	27.2	1.604	0.847–3.038	0.147
**HPV negative**									
IL-10 Low	55	68.4	1.000	Referent		48.0	1.000	Referent	
IL-10 High	29	68.7	1.323	0.521–3.361	0.556	53.1	1.237	0.610–2.507	0.555
**HPV positive**									
IL-10 Low	34	70.8	1.000	Referent		47.0	1.000	Referent	
IL-10 High	60	28.7	4.810	1.946–11.892	0.001	0.0	3.807	1.771–8.184	0.001

The interaction between HPV and IL-10: χ^2^ = 2.675, df = 1, P = 0.102 for OS; χ^2^ = 2.781, df = 1, P = 0.095 for RFS.

### A prognostic significance of IL-10 mRNA on OS and RFS was shown in female OSCC patients and OSCC patients who were non-smokers, non-drinkers, and non-betel quid chewers

As mentioned above, HPV infection and high IL-10 mRNA levels were more common in females, non-smokers, non-drinkers, and non-betel quid chewers. In addition, patients with HPV infection had higher levels of IL-10 mRNA expression than did patients without HPV infection. We therefore expected that the impact of IL-10 mRNA on OS and RFS would be predominantly observed in females, non-smokers, non-drinkers, and non-betel quid chewers. Multivariate logistic regression analysis indicated that the impact of IL-10 mRNA on both OS and RFS was indeed revealed in females, non-smokers, non-drinkers, and non-betel quid chewers, and not in males, smokers, drinkers, and betel-quid chewers (Table 4). The prognostic value of IL-10 mRNA on OS was also seen in males and smokers (HR, 2.14, 95% CI, 1.002–4.571, P = 0.049 for male; HR, 2.497, 95% CI, 1.160–5.373, P = 0.019 for smokers; Table 4). However, the HR value for OS and RFS in female and nonsmokers was higher than in male and smokers (Table 4). Therefore, these results suggest that the level of IL-10 mRNA expression is more feasible predictor of survival and relapse in female OSCC patients and in OSCC patients who are non-smokers, non-drinkers, and non-betel quid chewers.

**Table 4:** Multivariate analysis of the influence of various parameters on overall survival (OS) and relapse free survival (RFS) in oral cancer patients according to gender.

Parameter	Category	Case No.	OS				RFS			
			5-year survival (%)	HR	95% CI	P	5-year survival (%)	HR	95%CI	P
**Female**										
Stage	III+IV/I+II	25/45	36.3/72.0	3.393	1.389–8.289	0.007	10.6/58.3	2.844	1.441–5.614	0.003
HPV	Positive/Negative	56/14	51.9/83.9	1.715	0.484–6.081	0.404	26.9/84.6	3.764	1.111–12.749	0.033
IL-10	High/Low	38/32	24.5/76.3	3.610	1.345–9.689	0.011	0.0/65.0	9.258	3.272–26.196	<0.001
**Male**										
Stage	III+IV/I+II	50/58	22.7/81.9	3.707	1.622–8.471	0.002	0.0/60.3	4.017	2.112–7.641	<0.001
HPV	Positive/Negative	38/70	52.3/62.2	1.170	0.553–2.475	0.682	32.3/37.5	1.020	0.563–1.847	0.948
IL-10	High/Low	51/57	60.5/60.4	2.140	1.002–4.571	0.049	51.5/30.7	1.309	0.741–2.314	0.354
**Non-smoker**										
Stage	III+IV/I+II	24/49	35.8/74.0	2.791	1.108–7.031	0.029	5.9/58.5	2.459	1.200–5.036	0.014
HPV	Positive/Negative	53/20	53.8/77.9	0.732	0.213–2.517	0.620	27.9/63.8	0.988	0.376–2.596	0.981
IL-10	High/Low	39/34	32.1/75.9	3.121	1.011–9.635	0.048	0.0/60.6	5.894	2.081–16.695	0.001
**Smoker**										
Stage	III+IV/I+II	51/54	30.2/81.8	3.953	1.671–9.351	0.002	10.9/69.2	3.460	1.816–6.592	<0.001
HPV	Positive/Negative	64/41	50.0/71.1	1.279	0.595–2.748	0.528	31.1/51.9	1.121	0.612–2.052	0.712
IL-10	High/Low	50/55	56.1/55.1	2.497	1.160–5.373	0.019	46.8/31.9	1.428	0.803–2.539	0.225
**Non-drinker**										
Stage	III+IV/I+II	38/61	39.8/78.9	4.206	1.828–9.679	0.001	13.8/66.9	3.692	1.994–6.841	<0.001
HPV	Positive/Negative	64/35	55.9/77.1	1.335	0.480–3.711	0.579	33.6/61.5	1.290	0.614–2.710	0.502
IL-10	High/Low	47/52	45.5/75.9	2.995	1.292–6.939	0.011	23.5/59.0	2.643	1.389–5.029	0.003
**Drinker**										
Stage	III+IV/I+II	37/42	21.9/76.4	2.902	1.178–7.151	0.021	0.0/33.1	2.952	1.434–6.075	0.003
HPV	Positive/Negative	30/49	57.4/53.1	1.083	0.437–2.686	0.864	12.8/18.1	1.252	0.608–2.578	0.542
IL-10	High/Low	42/37	52.7/48.7	1.760	0.721–4.297	0.215	41.4/13.5	1.397	0.696–2.806	0.347
**Non-betel quid chewer**										
Stage	III+IV/I+II	34/46	34.3/64.2	3.523	1.446–8.583	0.006	11.0/64.7	2.911	1.456–5.818	0.003
HPV	Positive/Negative	60/20	51.3/68.0	0.598	0.192–1.864	0.376	27.3/70.9	1.238	0.459–3.341	0.673
IL-10	High/Low	40/40	25.9/70.2	5.395	1.886–15.431	0.002	0.0/58.1	7.002	2.640–18.571	<0.001
**Betel quid chewer**										
Stage	III+IV/I+II	41/57	27.3/81.6	3.118	1.322–7.356	0.009	0.0/54.6	3.459	1.819–6.579	<0.001
HPV	Positive/Negative	34/64	60.4/63.8	1.274	0.552–2.942	0.570	41.2/35.7	1.160	0.609–2.212	0.650
IL-10	High/Low	49/49	61.6/64.9	1.871	0.815–5.296	0.140	48.2/30.8	1.075	0.582–1.986	0.816

### Elevation of IL-10 expression by HPV16/18 E6 may be responsible for colony formation and migration capability in oral cancer cells

**Figure 2:**
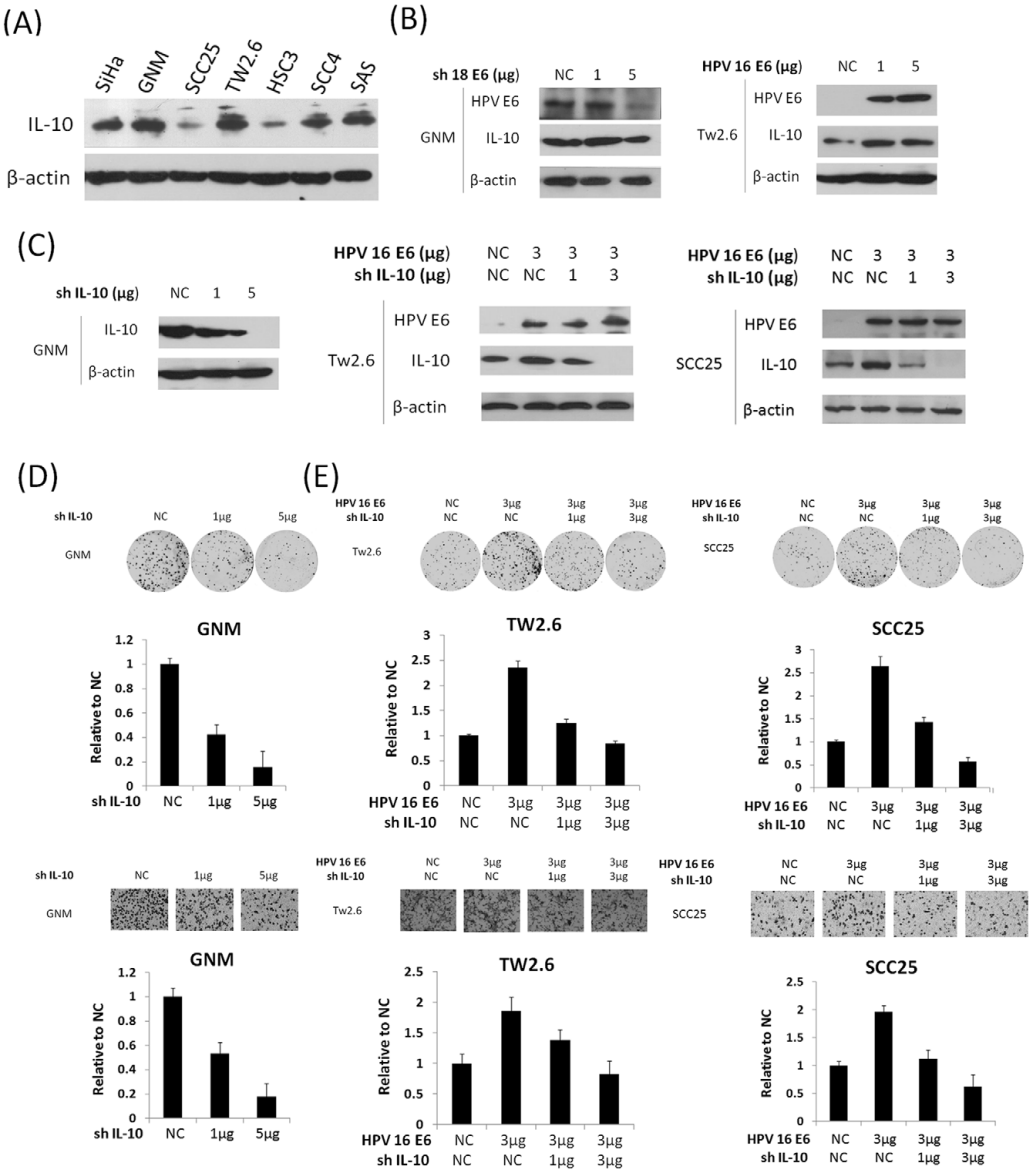
IL-10 expression increases associated with HPV16/18 E6 is responsible for the migration capability of OSCC cell lines. (A) IL-10 expression in a panel of OSCC cell lines was evaluated by Western blotting; (B) GNM cells were knocked down by transfection of shHPV18 E6 and Tw2.6 cells were transiently transfected with HPV16 E6 cDNA plasmid for 48 hrs. β-actin was used as a protein loading control; (C) GNM cells were knocked down by transfection of shIL-10. Tw2.6 and SCC25 cells were co-transfected with HPV16 E6 cDNA plasmid, and then E6 and IL-10 expression were determined by Western blotting. β-actin was used as a protein loading control; (D) The migration and colony formation capability of GNM cells with or without transfection of shIL-10 was evaluated by transwell migration assay; and (E) E6 transfected-Tw2.6 and -SCC25 cells were co-transfected with two doses of shIL-10, and then the migration and colony formation capability of both cells was evaluated by transwell migration assay.

As mentioned above, the prognostic significance of IL-10 mRNA expression was only observed in HPV-positive OSCC, not in HPV-negative OSCC. Therefore, we hypothesized that elevation of IL-10 by HPV infection could promote cell growth rate and migration capability. Western blotting analysis showed that HPV18-infected OSCC (GNM) had the highest IL-10 expression among all OSCC cells tested (Fig. 2A). The relatively low IL-10 expression in HPV16-infected SiHa cervical cancer cells was used as a positive control (Fig. 2A). To verify whether IL-10 could be elevated by HPV16/18 E6 oncoprotein, HPV18 E6 of GNM cells were knocked down by a small hairpin RNA (shRNA) (Fig. 2B). We also overexpressed an HPV16 E6 cDNA plasmid in HPV-negative TW2.6 and SCC25 cells (Fig. 2B). Western blotting analysis showed that IL-10 expression was decreased by E6-knockdown in GNM cells and increased by E6-overexpression in TW2.6 and SCC25 cells (Fig. 2B). Additionally, IL-10 expression was decreased by HPV16 or18 E6-knockdown in SiHa or HeLa cervical cancer cells and increased by HPV16 E6 overexpression C33A cervical cancer cells (Figure S1). These results suggest that HPV16/18 E6 infection may not only elevate IL-10 expression in OSCC cells but also observed in cervical cancer cells.

We next questioned whether IL-10 might be responsible for OSCC cell growth and migration capability. The colon formation and Boyden chamber assays indicated that the colony formation and migration capability decreased markedly in IL-10 knockdown GNM cells (upper panel for colony formation; lower panel for migration; Fig. 2C and 2D). In addition, the colony formation and migration capability was elevated by E6-overexpression in TW2.6 and SCC25 cells were eliminated by IL-10 knockdown (upper panel for colony formation; lower panel for migration; Fig. 2C and 2E). The representative colony formation and migration cell numbers with different treatments were shown in Figure 2D and 2E. The results from the cell model experiment may partially explain the observation that OSCC patients with high IL-10 expression had poorer survival and greater relapse with HPV-positive OSCC.

## Discussion

Our present study supports the finding that HPV-positive OSCC is a cancer that is distinct from HPV-negative OSCC; this distinction arises due to the association of HPV-negative OSCC with different etiological factors including cigarette smoking, alcohol drinking, and betel quid chewing. HPV16 has been considered as the predominant HPV subtype involved in oropharyngeal cancer [9], [10], [12]–[16], [27]. However, the infection rates for HPV18 were the same as for HPV16 (30.3%) in this study population (Table 1). More interestingly, HPV16 or HPV18 infection in these patients was exclusive because fewer than 10% patients (14 out of 178) had both types of HPV infection. Therefore, we speculated that HPV16 or HPV18 had an equal involvement in OSCC in the subset of Taiwanese patients.

HPV-infected oropharyngeal cancer has been shown to be more prevalent at the base of the tongue, in the pharynx, and in the tonsils, and less prevalent at the mobile or border of the tongue and buccal area [12], [16], [27], [33]. This phenomenon was explained by sexual behavior or marijuana use [42], [43]. However, the oral sexual behavior and marijuana use were seldom observed in the Taiwanese population [44], [45]. The infection rate of HPV16/18 was 65.2% and 41.2% for OSCC of tongue and buccal cavity, respectively (Table 1). This finding suggests that HPV16/18 infection may play an important role in patients with OSCC of tongue and buccal cavity who are non-smokers, non-drinkers, and non-betel quid chewers.

A high prevalence of OSCC patients who were females, non-smokers, non-drinkers, and non-betel quid chewers was noted in this study when compared with previous Taiwanese OSCC reports. This study population was similar to those reported previously that showed that the majority of Taiwanese male OSCC patients had one or more habit of cigarette smoking, alcohol drinking, and/or betel quid chewing (∼90%; Table S3). Interestingly, HPV-positive OSCC was more common in females than in males, which revealed that the existence of a different etiological profile between genders. The majority of female OSCC patients were non-smokers (84.9%), non-drinkers (62.6%), and non-betel quid chewers (82.5%); conversely, most male OSCC patients were smokers (92.4%), drinkers (89.9%), and betel quid chewers (95.9%).

Previous reports from the US and Sweden have also shown that HPV-positive OSCC in males was more common than in females [46]. However, in the present study, HPV-positive OSCC was more prevalent in females than in males. Therefore, our finding strongly supports a previous report that indicated a distinct risk factor profile for patients with HPV-positive OSCC when compared with HPV-negative OSCC patients.

A large body of evidence now reveals that HPV-positive OSCC has a more favorable outcome when compared with HPV-negative OSCC [7], [17]–[23]. However, HPV infection was not associated with patient outcomes in the present study population. Moreover, there is no difference in clinical outcome between HPV16- and HPV18-positive OSCC patients (p = 0.461 for OS; p = 0.403 for RFS). In current study, p16 expression used as a surrogate marker of HPV infection had a consistent prognostic value of previous reports [6], [16], [47], [48] showing that patients with p16 negative tumors had poorer outcome than those with p16-positive tumors (Table S4). This could be due to the bias of the study population, as our patients had one or more risk factors of cigarette smoking, alcohol drinking, and betel quid chewing. In the present study, 61 out of 178 patients (57 female and 4 male) had no risk factors for OSCC. To the best of our knowledge, this is the first study to use a similar number of risk and non-risk patients in a study population to investigate whether HPV-positive OSCC could be a distinct disease from the HPV-negative OSCC that is more common in smokers and drinkers.

HPV infection has been considered to be a favorable prognostic factor for OSCC, but no molecular evidence has yet supported this observation [7], [17]–[23]. In the present study, we provide *in vitro* cellular and *in vivo* human tumor evidence to indicate that IL-10 mRNA expression levels may independently predict the survival and relapse rates in patients with HPV-positive OSCC, but not in those with HPV-negative OSCC. This finding not only supports HPV-positive OSCC as a different disease from HPV-negative OSCC, but it also reveals that IL-10 may play a crucial role in the progression of HPV-positive OSCC.

It is conceivable that IL-10 may suppress T cell immunity, thereby resulting in a persistent HPV infection [2], [3], [32]. Consequently, the E6 oncoprotein will be expressed when HPV DNA becomes integrated into the host chromosome. The cell model data further showed that IL-10 expression elevated by E6 was responsible for colony formation and migration of OSCC cells. Promotion of lung cancer cell invasion by IL-10 was also reported previously [49]. Therefore, a positive feedback loop of IL-10 expression may contribute to HPV-positive OSCC progression.

## Supporting Information

Figure S1 **IL-10 expression was decreased by HPV16/18 E6-knockdown in SiHa or HeLa cells and increased by HPV16 E6 overexpression C33A cells.** SiHa cells were knocked down by transfection of shHPV16 E6 and HeLa cells were knocked down by transfection of shHPV18 E6. C33A cells were transfected with HPV16 E6 cDNA plasmid. HPV16/18 E6 and IL-10 expression was determined by Western blotting. β-actin was used as a protein loading control.
(TIFF)

Table S1 **Relationships between HPV infection and p16 expression in oral cancer patients.**
(DOC)

Table S2 **Relationships between IL-10 mRNA and clinical parameters in oral cancer patients.**
(DOC)

Table S3 **Relationships between gender and parameters in oral cancer patients.**
(DOC)

Table S4 **Multivariate analysis of the influence of HPV 16/18 expression and p16 expression on overall survival (OS) and relapse free survival (RFS) in oral cancer patients.**
(DOC)
