# Supplementary figures and images for: Differential Impact of IL-10 Expression on Survival and Relapse between HPV16-Positive and -Negative Oral Squamous Cell Carcinomas
Source: PLoS One. 2012 Oct 31;7(10):e47541. doi: 10.1371/journal.pone.0047541 (PMC3485273; doi:10.1371/journal.pone.0047541)

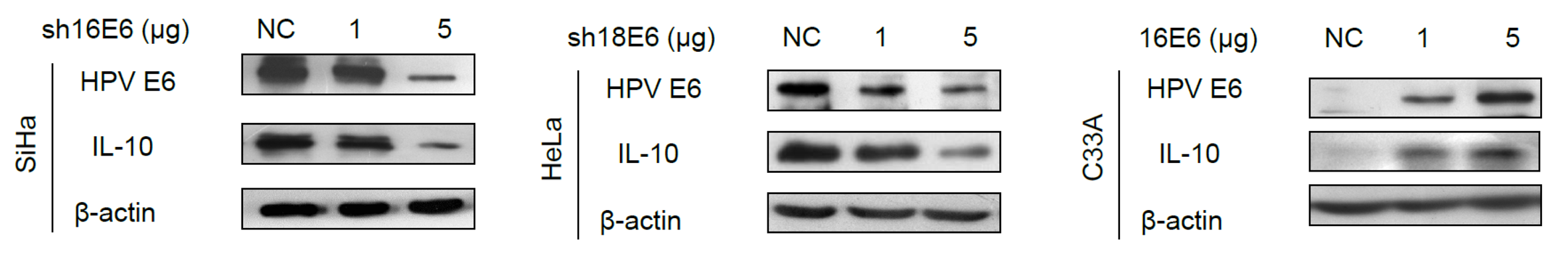

Supplement: Figure S1 — IL-10 expression was decreased by HPV16/18 E6-knockdown in SiHa or HeLa cells and increased by HPV16 E6 overexpression C33A cells. SiHa cells were knocked down by transfection of shHPV16 E6 and HeLa cells were knocked down by transfection of shHPV18 E6. C33A cells were transfected with HPV16 E6 cDNA plasmid. HPV16/18 E6 and IL-10 expression was determined by Western blotting. β-actin was used as a protein loading control. (TIFF) [file pone.0047541.s001.tiff]
