# Supplementary material for: Differential Impact of IL-10 Expression on Survival and Relapse between HPV16-Positive and -Negative Oral Squamous Cell Carcinomas
Source: PLoS One. 2012 Oct 31;7(10):e47541. doi: 10.1371/journal.pone.0047541 (PMC3485273; doi:10.1371/journal.pone.0047541)
Supplement: Table S1 — Relationships between HPV infection and p16 expression in oral cancer patients. (DOC) [file pone.0047541.s002.doc]

| Supplementary Table 1. Relationships between HPV infection and p16 expression in oral cancer patients. | | | | |
| --- | --- | --- | --- | --- |
| Parameters | Case No. | HPV 16 | HPV 18 | HPV 16/18 |
| p16 |  |  |  |  |
| Low | 78 | 15 (19.2)* | 13 (16.7)* | 27 (34.6)* |
| High | 69 | 28 (40.6) | 24 (34.8) | 44 (63.8) |

One hundred and fourty-seven of 178 tumors were available for detection of p16 expression by immunohistochemistry.

* p < 0.05.
