# Supplementary material for: Differential Impact of IL-10 Expression on Survival and Relapse between HPV16-Positive and -Negative Oral Squamous Cell Carcinomas
Source: PLoS One. 2012 Oct 31;7(10):e47541. doi: 10.1371/journal.pone.0047541 (PMC3485273; doi:10.1371/journal.pone.0047541)
Supplement: Table S2 — Relationships between IL-10 mRNA and clinical parameters in oral cancer patients. (DOC) [file pone.0047541.s003.doc]

| Supplementary Table 2. Relationships between IL-10 mRNA and clinical parameters in oral cancer patients. | | | | |
| --- | --- | --- | --- | --- |
|  |  | IL-10 mRNA | |  |
| Parameters | Case No. | Low (%) | High (%) | P value |
| Age |  |  |  |  |
| <56 | 105 | 59 (56.2) | 46 (43.8) | 0.048 |
| ≥56 | 73 | 30 (41.1) | 43 (58.9) |  |
| Gender |  |  |  |  |
| Female | 70 | 32 (45.7) | 38 (54.3) | 0.357 |
| Male | 108 | 57 (52.8) | 51 (47.2) |  |
| Smoking |  |  |  |  |
| No | 73 | 34 (46.6) | 39 (53.4) | 0.446 |
| Yes | 105 | 55 (52.4) | 50 (47.6) |  |
| Drinking |  |  |  |  |
| No | 99 | 52 (52.5) | 47 (47.5) | 0.451 |
| Yes | 97 | 37 (46.8) | 42 (53.2) |  |
| Betel nut |  |  |  |  |
| No | 80 | 40 (50.0) | 40 (50.0) | 0.999 |
| Yes | 98 | 49 (50.0) | 49 (50.0) |  |
| Stage |  |  |  |  |
| I | 65 | 37 (56.9) | 28 (43.1) | 0.396 |
| II | 38 | 20 (52.6) | 18 (47.4) |  |
| III | 23 | 10 (43.5) | 13 (56.5) |  |
| IV | 52 | 22 (42.3) | 30 (57.7) |  |
| T value |  |  |  |  |
| T1 | 78 | 42 (53.8) | 36 (46.2) | 0.654 |
| T2 | 57 | 29 (50.9) | 28 (49.1) |  |
| T3 | 7 | 3 (42.9) | 4 (57.1) |  |
| T4 | 36 | 15 (41.7) | 21 (58.3) |  |
| N value |  |  |  |  |
| N0 | 128 | 69 (53.9) | 59 (46.1) | 0.249 |
| N1 | 25 | 10 (40.0) | 15 (60.0) |  |
| N2 | 25 | 10 (40.0) | 15 (60.0) |  |
| Tumor site |  |  |  |  |
| Tongue | 92 | 43 (46.7) | 49 (53.3) | 0.460 |
| Buccal | 68 | 38 (55.9) | 30 (44.1) |  |
| Other | 18 | 8 (44.4) | 10 (55.6) |  |
