# Supplementary material for: Differential Impact of IL-10 Expression on Survival and Relapse between HPV16-Positive and -Negative Oral Squamous Cell Carcinomas
Source: PLoS One. 2012 Oct 31;7(10):e47541. doi: 10.1371/journal.pone.0047541 (PMC3485273; doi:10.1371/journal.pone.0047541)
Supplement: Table S3 — Relationships between gender and parameters in oral cancer patients. (DOC) [file pone.0047541.s004.doc]

| Supplementary Table 3. Relationships between gender and parameters in oral cancer patients. | | | | |
| --- | --- | --- | --- | --- |
|  |  | Gender | |  |
| Parameters | Case No. | Female (%) | Male (%) | P value |
| Smoking |  |  |  |  |
| No | 73 | 62 (84.9) | 11 (15.1) | <0.001 |
| Yes | 105 | 8 (7.6) | 97 (92.4) |  |
| Drinking |  |  |  |  |
| No | 99 | 62 (62.6) | 37 (37.4) | <0.001 |
| Yes | 79 | 8 (10.1) | 71 (89.9) |  |
| Betel-quid chewing |  |  |  |  |
| No | 80 | 66 (82.5) | 14 (17.5) | <0.001 |
| Yes | 98 | 4 (4.1) | 94 (95.9) |  |
| Combination |  |  |  |  |
| None^1^ | 61 | 57 (93.4) | 4 (6.6) | <0.001 |
| One or more^2^ | 117 | 13 (11.1) | 104 (88.9) |  |

^1^Patients did not have the habits of cigarette smoking, drinking, and betel quid chewing.

^2^Patients had one or more habits including cigarette smoking, drinking, and betel quid chewing.
