# Supplementary material for: Differential Impact of IL-10 Expression on Survival and Relapse between HPV16-Positive and -Negative Oral Squamous Cell Carcinomas
Source: PLoS One. 2012 Oct 31;7(10):e47541. doi: 10.1371/journal.pone.0047541 (PMC3485273; doi:10.1371/journal.pone.0047541)
Supplement: Table S4 — Multivariate analysis of the influence of HPV 16/18 expression and p16 expression on overall survival (OS) and relapse free survival (RFS) in oral cancer patients. (DOC) [file pone.0047541.s005.doc]

| Supplementary Table 4. Multivariate analysis of the influence of HPV 16/18 expression and p16 expression on overall survival (OS) and relapse free survival (RFS) in oral cancer patients. | | | | | | | | | | |
| --- | --- | --- | --- | --- | --- | --- | --- | --- | --- | --- |
| **p16 expression** | Case No. | OS | | | |  | RFS | | | |
|  |  | 5-year survival (%) | HR | 95% CI | P |  | 5-year survival (%) | HR | 95%CI | P |
| **All cases** |  |  |  |  |  |  |  |  |  |  |
| High | 69 | 71.7 | 1.000 | Referent |  |  | 52.6 | 1.000 | Referent |  |
| Low | 78 | 53.5 | 1.584 | 0.790-3.178 | 0.195 |  | 21.1 | 1.873 | 1.138-3.084 | 0.014 |
| **HPV-negative** |  |  |  |  |  |  |  |  |  |  |
| High | 25 | 81.8 | 1.000 | Referent |  |  | 65.0 | 1.000 | Referent |  |
| Low | 51 | 54.7 | 1.393 | 0.443-4.379 | 0.571 |  | 31.0 | 1.559 | 0.658-3.693 | 0.313 |
| **HPV-positive** |  |  |  |  |  |  |  |  |  |  |
| High | 44 | 65.1 | 1.000 | Referent |  |  | 47.3 | 1.000 | Referent |  |
| Low | 27 | 51.0 | 2.097 | 0.831-5.293 | 0.117 |  | 8.5 | 3.292 | 1.694-6.397 | <0.001 |
